# Development of the Dual-Beam Ion Irradiation Facility for Fusion Materials (DiFU)

**DOI:** 10.3390/ma16031144

**Published:** 2023-01-29

**Authors:** Tonči Tadić, Toni Dunatov, Stjepko Fazinić, Donny Domagoj Cosic, Milko Jakšić, Zdravko Siketić, Milan Vićentijević, Wataru Kada, Christopher D. Hardie

**Affiliations:** 1 Ruđer Bošković Institute, Bijenička 54, 10000 Zagreb, Croatia; 2 Department of Electric Engineering, Gunma University, 4 2 Aramakimachi, Maebashi 371-8510, Gunma, Japan; 3 UK Atomic Energy Authority, Culham Science Centre, Oxfordshire, Abingdon OX14 3DB, UK

**Keywords:** fusion materials, ion irradiation, dual-beam ion irradiation facility

## Abstract

The Dual-beam ion irradiation facility for Fusion materials (DiFU) has been developed and installed at the Ruđer Bošković Institute with the purpose to perform irradiation of samples of fusion materials by one or two ion beams. Ion beams are delivered to the DiFU chamber by a 6 MV EN Tandem Van de Graaff and a 1 MV HVE Tandetron accelerator, enabling irradiation of areas up to 30 × 30 mm^2^. The sample holder enables the three-dimensional positioning of samples that can be irradiated while being heated, cooled, or kept at room temperature. Ion fluxes are measured indirectly by the insertion of two large Faraday cups. Besides, the ion flux is monitored continuously by two sets of horizontal and vertical slits, which, in turn, define the limits of the irradiation area on the sample. Sample temperature and conditions during irradiation are additionally monitored by a set of thermocouples, an IR camera, and a video camera. Particular care is dedicated to the mitigation of carbon contamination during ion irradiation.

## 1. Introduction

Ion irradiation by single, dual, or triple ion beam facilities [1,2,3,4,5,6,7] can generate high radiation damage doses within practical timescales (e.g., hours) and is therefore a promising method for the selection of radiation-resistive candidate materials for future fusion power plants and next generation of fission power plants. The key point here is that charged particle irradiation can be used to investigate the effects of neutron radiation damage. A characteristic advantage of charged-particle irradiation experiments in comparison to neutron irradiation experiments [8] is precise control over most of the important irradiation conditions such as dose, dose rate, temperature, and quantity of gases present in the vacuum irradiation environment. An additional advantage of charged-particle irradiation experiments in comparison to neutron irradiation experiments is the lack of induced radio-activation of specimens and, in general, a substantial acceleration of irradiation time, from years to hours, to achieve comparable damage [9,10,11,12,13,14] as typically measured in displacements per atom (dpa) [8]. An important application of such experiments is the investigation of radiation effects in not-yet-existing environments, such as fusion reactors [15,16]. This paper presents: (i) the design and development of the Dual-beam ion irradiation facility for FUsion materials (DiFU) with the purpose to perform irradiation of fusion-related samples by one or two ion beams; (ii) protocols and procedures adopted at the DiFU for ion irradiations of nuclear material samples.

DiFU has been developed at the Tandem Accelerator Facility of the Ruđer Bošković Institute in Zagreb, Croatia. It allows the irradiation of samples by one or simultaneously by two ion beams. Namely, the RBI accelerator facility is one of only a dozen world laboratories equipped with the possibility to irradiate materials by simultaneous irradiation with two ion beams (dual-ion beam concept), using two accelerators: 6 MV Tandem Van de Graff and 1 MV Tandetron. The DiFU facility was commissioned in 2019, with improvements introduced every year since.

## 2. Materials and Methods

### 2.1. Accelerators and Ion Sources

The RBI Tandem Accelerator Facility has been regularly used for ion beam analysis and materials modification studies, including the investigation of materials relevant to nuclear energy [17,18,19]. It has been designed in a way to maximize the possibility of delivering a variety of ion beams from any of the two available accelerators into the majority of end stations (7 out of 9). The span of ion beam energies that could be delivered to these end stations starts from just 100 keV from the small tandem accelerator up to several tens MeVs for multi-charged heavy ions from the larger EN tandem accelerator. Currently, this accelerator operates stably at terminal voltages between 0.5 and 4.0 MV. The kinetic energy of the ion beam is kept constant durion irradiation using calibrated 90-degrees analytical magnets at both accelerators and magnetic field gauges. The utilization rate during ion beam transport is several percent.

**Figure 1:**
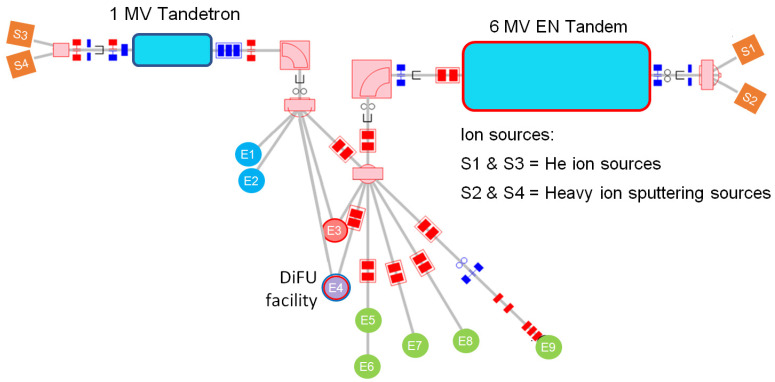
Beam lines at RBI’s Accelerator Centre; E1 = PIXE/RB/NRA chamber; E2 = in-air PIXE end station; E3 = dual-microbeam chamber; E4 = DiFU chamber; E5 = TOF-ERDA chamber; E6 = MeV SIMS chamber; E7 = RBS/PIXE channeling chamber; E8 = NRA chamber; E9 = heavy ion microbeam chamber.

Another design feature of the RBI accelerator facility is its capability to deliver simultaneously two ion beams into the single scattering chamber. As it is seen in Figure 1, there are two such dual beam end stations (E3 and E4), one of which, the DiFU chamber (E4), is described in this paper.

Particularly important is the capability that both accelerators can provide a variety of ion species from their ion sources. EN tandem Van de Graaff is equipped with multicathode NEC SNICS II sputtering ion source used for the majority of ion species and with NEC Alphatross RF source with charge exchange channel that is used mainly for He ion beams. 1 MV Tandetron is equipped with a single cathode NEC SNICS sputtering ion source for the majority of ions (including hydrogen) and HVE duoplasmatron with a charge exchange channel used mostly for helium ions.

To irradiate materials at similar sample depths, dual beam irradiations at the DiFU facility are done by higher energy heavy ions from the EN tandem Van De Graaff and by protons or He ions from 1 MV Tandetron. Both accelerators also enable single-ion beam irradiation. Proton and alpha-particle irradiations are frequently used to inject hydrogen and helium into specimens to simulate the production of hydrogen and helium in investigated material as an effect of neutron irradiations where hydrogen and/or helium would be produced in transmutation reactions. Hydrogen and helium injection may be completed before heavy particle irradiation begins. It may also proceed incrementally during interruptions in the heavy particle irradiation or simultaneously with heavy particle irradiation. The last case is the most desirable as it gives the closest simulation to neutron irradiation effects [8,20]. Besides protons and He ions, various ions have been used for irradiation at DiFU: Fe, Cu, W, I, Au, as well as Li, C, O, Si and Ge.

### 2.2. Ion Beam Handling

**Figure 2:**
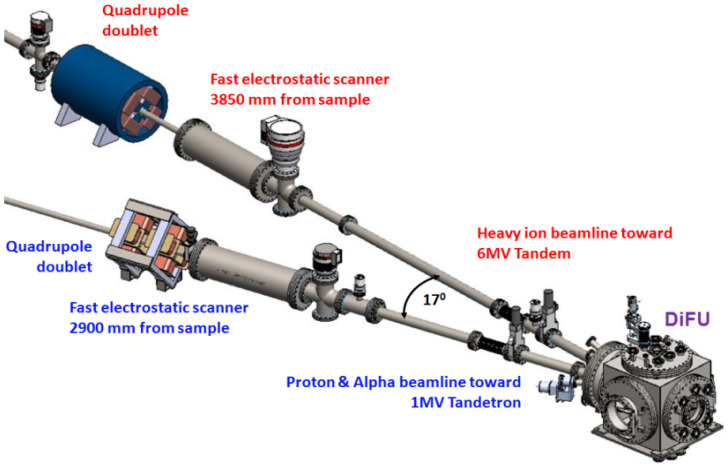
Beamlines to the DiFU’s irradiation chamber.

The DiFU irradiation chamber is connected to accelerators by two beam lines shown in Figure 2. Through the beamlines, each ion beam reaches a sample mounted on a holder in the irradiation chamber at an angle of 8.5° to the normal sample surface.

To achieve ion dose rate homogeneity over the irradiation area, both beamlines are equipped with fast electrostatic scanners that enable beam scanning in the kHz range. A typical ion beam spot at DiFU before starting the ion beam scanning covers about 70% of the irradiation area.

Two dedicated pairs of electrostatic scanners, made by Danfysik, are installed at both beamlines. Scanners are designed to achieve deflection of at least ±20 mm at the sample position, with the plate lengths of 250 mm separated by 30 mm and with ±10 kV maximal voltage difference between the plates. The theoretical maximal ion beam scanned the area at the sample position is 40 × 40 mm^2^. A dedicated programmable Scanning Control Card and related software have been developed at the RBI for the purpose of synchronized simultaneous scanning of both ion beams. Adjustment of scanning areas is enabled separately for each of the two ion beams. Matsusada 10B10 High-voltage High-frequency Amplifiers are employed for the conversion of the Scanning Control Card’s pulses from volt to kiloVolt range which is then sent to the electrostatic Danfysik scanners. The scanning pattern consists of at least 10 evenly spaced positions on the horizontal axis, as well as minimal 10 positions on the vertical axis. Since the minimal dwell time of ion beams at each position could be as low as 10 μs, it gives a theoretical maximal scanning frequency of 10 kHz for horizontal or vertical scanning of ion beams.

### 2.3. DiFU’s Irradiation Chamber

**Figure 3:**
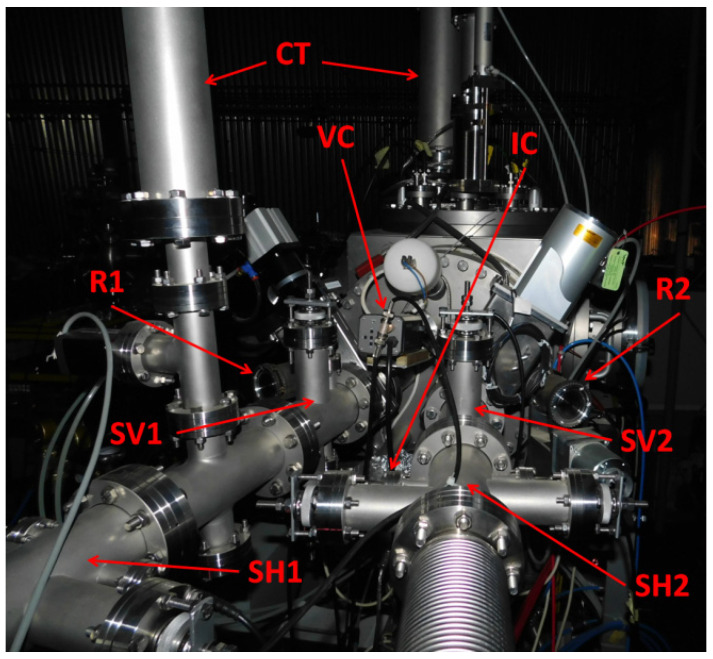
DiFU’s chamber’s front flange; CT—Liquid Nitrogen Cold Traps; R1 & R2—Magnetic Coupling Rotators for ion beam energy degraders; SH1 & SH2—Horizontal Slits; SV1 & SV2—Vertical Slits; VC—Video Camera; IC—Infra-Red Camera.

The DiFU irradiation chamber is designed as a cube with the size of 48 × 48 × 48 cm^3^. At the beam entrance side of the chamber, two beamlines finish with a set of beam-defining apertures. This side also accommodates the ports with axes for the beam degraders, sample viewer cameras, and scattered particle detectors (Figure 3). All other chamber sides are equipped with a large number of various ports used for auxiliary equipment, different feed troughs, gauges, etc., and also for installation of equipment required by users for irradiations of their samples. A good example of this relatively unique versatility is the installation of the external user equipment to irradiate samples with a temperature gradient, where a range of irradiation temperatures could then be investigated from a single irradiation experiment [21].

**Figure 4:**
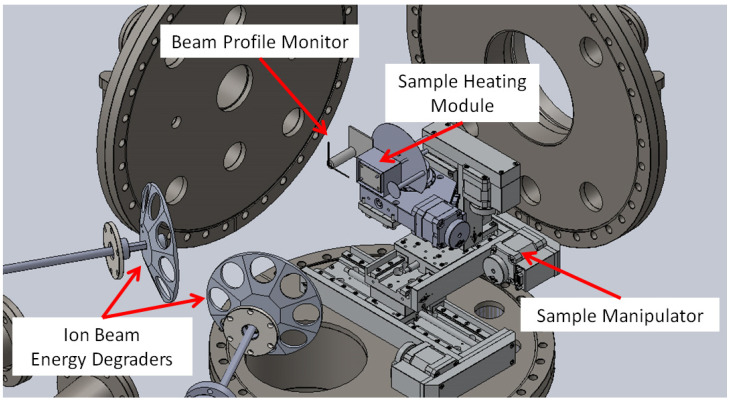
Position of Beam Profile Monitor, Sample Heating Module, and Ion Beam Energy Degraders within DiFU’s irradiation chamber.

The sample for irradiation can be precisely positioned within DiFU’s chamber by Sample Manipulator (Figure 4), at which two different sample holders could be placed, one for ion irradiation of samples at room temperature or the other one for irradiations at elevated temperatures. The sample manipulator allows three degrees of freedom for sample movements within these limits: Left-Right = 120 mm; Up-Down = 50 mm; Back-Forth = 100 mm.

### 2.4. Sample Heating

**Figure 5:**
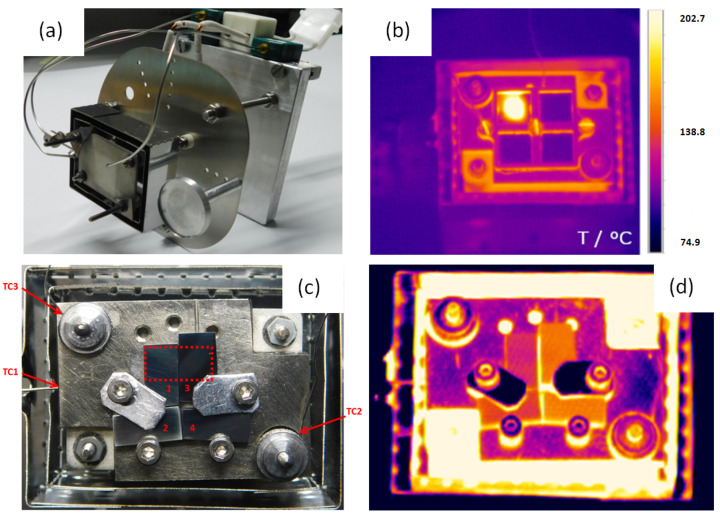
(**a**) DiFU’s Sample Heating Module; (**b**) IR image of Sample Heater at, with samples attached to Small Samples’ Adapter. Beam-induced heating of thin iron foils is visible; (**c**) Small Samples’ Adapter with four samples attached at stainless steel backplate, with marked irradiation area 10 × 5 mm^2^ and indicated position of three thermocouples (small samples are marked with numbers 1–4); (**d**) The same set of four samples heated at 600 °C.

Heating of a sample within the DiFU chamber is realized by a sample heating module, based on Tectra Boralectric Ohmic Heater HTR1001 with an effective heating area of 30 × 20 mm^2^, and 2 mm thickness (Figure 5a). The sample heating module is equipped with molybdenum heat shields and fused quartz to check the ion beam scanning area. A K-type shielded thermocouple made by Omega is attached to a heater for temperature control and sample temperature is controlled by two additional Omega thermocouples of the same type, placed at sample edges. The heater, made of Pyrolytic Boron Nitride and Pyrolytic Graphite, is commonly used at temperatures up to 750 °C. The heaters power supply is a Tectra HC3500-LV with PID controller, which maintains a stable sample temperature within ±1 °C.

Irradiation of samples is commonly done with very low ion beam flux intensity, in prolonged irradiation mode. In this way, ion beam flux intensity is generally kept below levels which may result in sample heating. Effects of sample heating can be estimated before the experiment [22,23,24,25], however, they are typically negligible unless the samples are very thin.

The low heater volume enables rapid cooling and very high heating rates. Holders for smaller samples are commonly prepared to user requirements.

Temperature distribution over a sample is measured by Optris PI640 Infrared (IR) camera, with a position resolution of 0.4 mm at the sample surface, temperature range from −20 °C to 900 °C, and temperature resolution of 75 mK. The infrared camera is installed at an infrared transparent ZnSe CF38 window procured from Kurt J. Lesker. An example of heating measurement from the IR camera is shown in Figure 5b,c.

The IR camera used with ZnSe IR-transparent window is tuned for the temperature range of −20–900 °C. Thermocouples used are K-type which are suitable in a range from room temperature to 1200 °C.

### 2.5. Ion Flux Estimation and Constant Ion Flux Monitoring

Precise dose measurement is of key importance for the ion irradiation of materials. Irradiation of a heated sample, however, requires indirect ion beam flux measurement and indirect checking of the irradiation area on the sample surface. This holds for single ion irradiation, as well as for simultaneous irradiation by two ion beams. The irradiation area at DiFU is defined by pairs of adjustable slits within each beamline.

The theoretical irradiated area is pre-set by the positioning of horizontal and vertical slits. The size and position of the actual irradiated area are then checked by ionoluminescence at fused quartz, with a millimeter grid on. The accuracy of the ionoluminescence method has been tested by irradiation and darkening of Kapton foil, as well as by Beam Profile Monitor placed at the sample manipulator.

**Figure 6:**
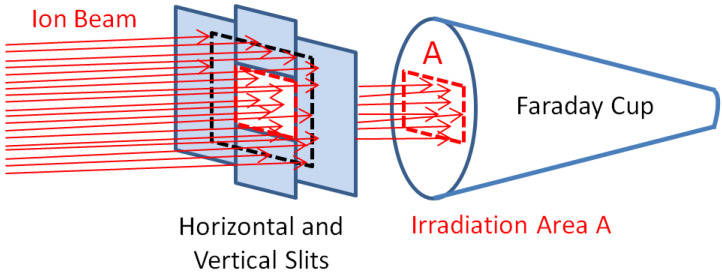
Definition of irradiation area A by slits in relation to Faraday Cup setup at the beamline. Red dotted line marks irradiation area A. Black dotted line marks limits of area swept by scanning ion beam.

Both methods used have not shown a discrepancy in the irradiated area determined by ionoluminescence at fused quartz. The slits, furthermore, enable continuous precise measurements of ion dose fluctuations for each beamline, while a large Faraday cup (FC), shown in Figure 6 allows for precise ion dose measurement. The maximal opening of the slit setups is ±15 mm vertical and ±15 mm horizontal. A circular opening of Faraday cups (FC) is 35 mm in diameter. A suppression electrode of the same diameter is positioned in front of the FC and is kept at −300 V during irradiations, to mitigate secondary electron emission from FC. The maximum opening of slits is always smaller than the opening of FC, to ensure that the entire ion flux passing through slits is collected in the FC; this enables precise estimation of the ion flux delivered to the sample.

It is obvious that the ion flux cannot be measured continuously by FC. Therefore, ion flux measurement is performed in regular intervals during irradiation by inserting the FC in the ion beam for about 10 to 15 s to ensure averaging of ion flux over time. All the time during irradiation ion current is collected at every slit and in 3 s bins (which includes 1 s for re-switching and 2 s for current collection). Slits are without electron suppressor electrodes, i.e., current readings from slits are relative. Together with periodical FC readings, they help to continuously estimate the dose rate delivered to a sample during irradiation. Ion beam currents, collected by slits or by FCs are measured by a Keithley 6485E Digital Multimeter and by a Keithley 7001 Module Multiplexer, using dedicated software developed by RBI, for switching between FCs and XY slits.

In all experiments SRIM has been used in the Kinchin-Pease (KP) mode, to calculate the total fluence needed to reach a certain dpa value [26,27,28]. The other parameter needed is the displacement energy of the material [16] taken from tabulated values [8], or agreed with the user.

### 2.6. Homogeneity of Irradiation

The ion flux profile at DiFU is estimated by a Beam Profile Monitor (BPM), placed at the Sample Manipulator, in form of an insulated L-shaped flat copper wire, with L sides 1 mm wide and 40 mm long, as shown schematically in Figure 4. The advantage of this configuration of BPM is that it checks the ion flux profile at the sample position, rather than at the beamline entry point in front of the irradiation chamber. The BPM utilizes precise positioning of the sample manipulator for measuring the ion beam current from the L-shaped flat copper wire while it crosses the ion beam, separately for the horizontal and vertical profile. Only one ion beam can be profiled at a time, while another ion beam is deflected. Secondary electron suppression is not used for the L-shaped flat copper wire so ion beam currents are given in relative units.

**Figure 7:**
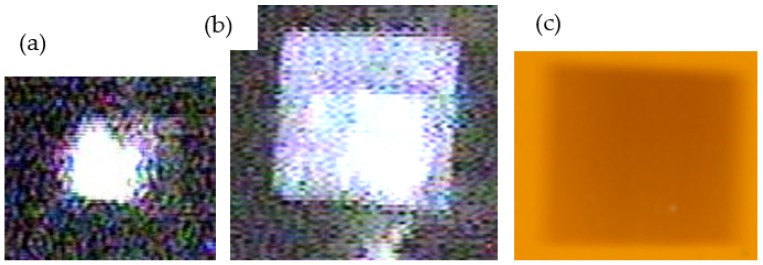
(**a**) Stationary 10 MeV Fe^+4^ 160 nA ion beam spot at fused quartz; (**b**) The same Fe^+4^ ion beam “wobbled” over irradiation area 15 × 15 mm^2^ (lighter part of the image is a result of reflection of illumination light); (**c**) Darkening of Kapton foil with the same “wobbled” Fe^+4^ ion beam shown in the same scale.

Particular care is taken to ensure that the ion beam dose is equal at all locations on the irradiated sample. Namely, the ion beam spot prior to “beam wobbling” commonly covers over 50% of the irradiated area, and by high-frequency “beam wobbling” the dose is equalized over the irradiated area, as shown below (Figure 7):

Dose homogeneity is checked by ionoluminescence at fused quartz and by equal readings on left and right horizontal slits and up and down vertical slits, as shown schematically in Figure 6.

### 2.7. Adjusting of Ion Energy

The ion energy is defined and kept constant during ion irradiation by the 90° analyzing magnets after each of the accelerators, enabling accurate selection of the desired ion energy. The relationship between magnetic field and energy is calibrated using known resonances as explained in ref. [29].

**Figure 8:**
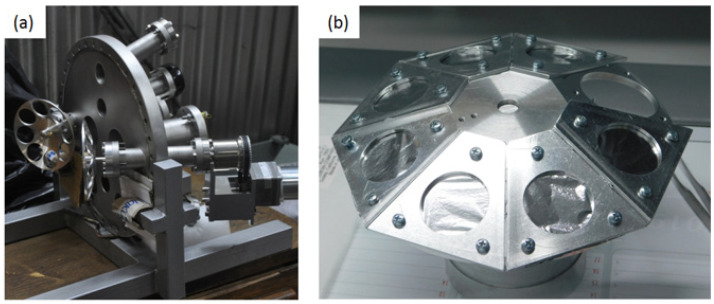
(**a**) Position of ion beam energy degraders at the front flange of DiFU chamber; (**b**) Ion beam energy degrader with aluminum foils attached.

Multi-ion-beam facilities [1,5,6] often use thin aluminum foils to degrade ion beam energies during irradiation by utilizing the stopping of ions in aluminum. In such a way the damage-depth profile in the irradiated sample can be adjusted, as well as the implantation-depth profiles of helium and hydrogen. Aluminum foils used for this purpose are commonly placed on rotating or oscillating foil holders, commonly referred to as “ion beam energy degraders”, as shown in Figure 8.

The ion beam energy degraders in DiFU are made from a single aluminum block, which ensures good conductivity and transfer of heat induced by ion beams within the foils. Ion beam energy degraders are octagonal, 15 cm in diameter, with seven foil-covered positions and one empty position. Each position has a circular hole of 30 mm in diameter. Aluminum foils of 0.8–6.0 μm thickness are commonly used, with exact arrangements determined by user requirements. Ion beam energy degraders and respective foils are positioned at 250 mm from the irradiated sample. Ion beam energy degraders are fixed to rotating shafts and placed at 20 degrees to the respective ion beam. Two rotational magnetic-coupling vacuum feedthroughs Kurt J. Lesker MD-35, equipped with a side-mounted DC electric motor, are installed on the front flange of the DiFU chamber. The maximum rotation speed of the foil degraders is 200 rpm.

Effects of ion beam spread, caused by ion Multiple Scattering (MS) in degrader foils, have to be taken into account when ion beam energy degraders are used [30]. The angular spreads of heavy ions in Al foils could be modeled using Sigmund and Winterborn [31] model of MS angular distributions and their approximation by Pseudo-Voigt distributions [32,33]. The angular spread of protons and He ions due to MS in aluminum foils could be simulated as well. Ion beam energy degraders at DiFU are commonly only used for proton or alpha-particle beams.

### 2.8. Mitigation of Carbon Contamination in the Vicinity of the Irradiated Sample

Carbon contamination of samples during irradiation can be a serious problem, including the deposition of hydrocarbons at irradiated sample surfaces and the implantation of carbon (as well as nitrogen and oxygen) in the sample layers below the surface. Such effects could alter the chemical composition in the irradiated regions of a sample and may have an impact on post-irradiation examination [34,35,36,37,38,39,40]. Regardless of the mechanisms involved, carbon deposition at the sample surface has its source in residual gases in the DiFU chamber and beamlines. Vacuum systems equipped with turbo-molecular pumps and copper gasket seals, such as that used for the DiFU chamber and related beamlines, have residual gasses composed of 10% (CH_4_+H_2_O), 35% (CO+N_2_) and 5% CO_2_, while remaining gasses are mostly hydrogen and oxygen [41]. In practical terms, at room temperature and vacuum pressure of 3 × 10^−8^ mbar in the DiFU chamber, there would be about 5 × 10^7^ cm^−3^ of carbon atoms suitable for deposition onto the irradiated sample at any time. In reality, however, various heavier hydrocarbon gasses could be present, as well.

A key pre-condition for the mitigation of hydrocarbon implantation at sample surfaces is the maintenance of a high vacuum in the chamber and in associated beamlines. Pumping stations on the beamlines enable vacuum pressures of 3 × 10^−8^ mbar or better, while in the irradiation chamber, the vacuum pressure is also kept at about 3 × 10^−8^ mbar. The DiFU chamber and beamlines are baked regularly and in particular after major repairs or replacements of components in the chamber. In addition, plasma cleaning is applied in the chamber and to every component to be installed. Since the DiFU chamber is not equipped with a vacuum load lock it is commonly flushed with nitrogen gas for every intervention, including sample insertion or removal.

Liquid nitrogen (LN2) cold traps are installed at the DiFU chamber and at beamlines, which cooled with liquid nitrogen to 173 K serve as a getter for contaminant molecules. Cold traps are filled with LN2 prior to irradiation. By using LN2 cold traps at the beamlines, the vacuum at the beamlines is slightly better than in the DiFU chamber, thus reducing the possible jet stream of residual gasses from beamlines toward the sample.

**Figure 9:**
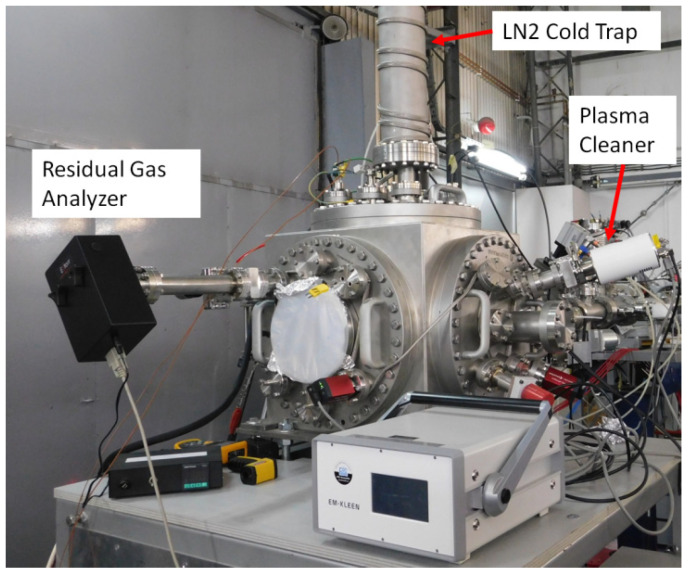
DiFU chamber with LN2 cold trap, TECTRA ExTorr Residual Gas Analyzer Residual Gas Analyzer, and EM-KLEEN Plasma Cleaner.

Finally, a TECTRA ExTorr Residual Gas Analyzer (RGA) is installed at the DiFU chamber to monitor partial residual gasses pressure present before, during, and after irradiations. This instrument is also used to check the efficiency of various carbon contamination mitigation methods, such as cleaning the samples and the vacuum chamber with oxygen plasma [34,35,37]. For this purpose, an EM-KLEEN Plasma Cleaner is used, as shown in Figure 9.

## 3. Results and Discussion

Heavy ions recently used were a 10 MeV Fe^3+^ ion beam and 12 MeV Au^5+^ ion beam for irradiation of steel samples and alumina-coated steel samples to 10 dpa. Total irradiation damage dose was achieved during 43.3 h irradiation with a total fluence of 3.7 × 10^16^ Fe^3+^ ions/cm^2^ and 24.5 h irradiation with a total fluence of 9.5 × 10^16^ Au^5+^ ions/cm^2^, respectively, with a damage dose rate of 6.52 × 10^−5^ dpa/s and 1.15 × 10^−4^ dpa/s, respectively. Dual-beam simultaneous irradiation of steel samples with iron and helium ions was performed using a 10 MeV Fe^4+^ ion beam of 169 nA ion current in a 15 × 15 mm^2^ irradiation area and 1.2 MeV He^2+^ ion beam of 4.8 nA ion current in the same irradiation area.

### 3.1. Testing of Ion Flux and Flux Fluctuations

**Figure 10:**
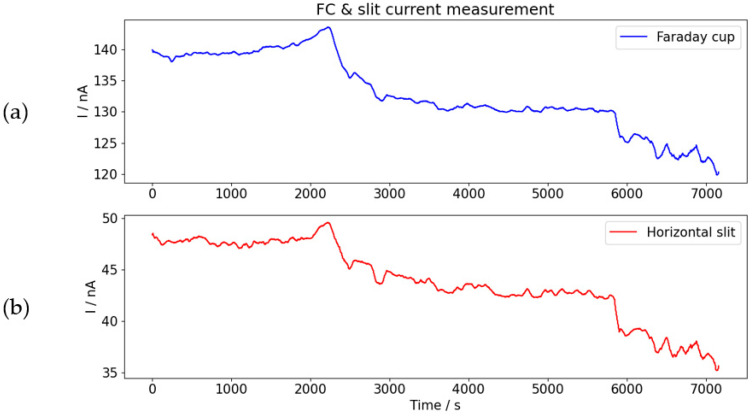
Comparison of ion current readings from Faraday cup (**a**) with ion current reading from slits (**b**).

A comparison of the measurements produced by FC and slits is shown in Figure 10. for Carbon ions of 0.5 MeV, scanned over an irradiation area of 10 by 10 mm^2^.

As shown in Figure 10, although slits are without electron suppressor electrodes, i.e., current readings from slits are relative and proportional to ion flux, they are good indicators of trends and changes in ion flux.

### 3.2. Determination of Ion Beam Profile Homogeneity

**Figure 11:**
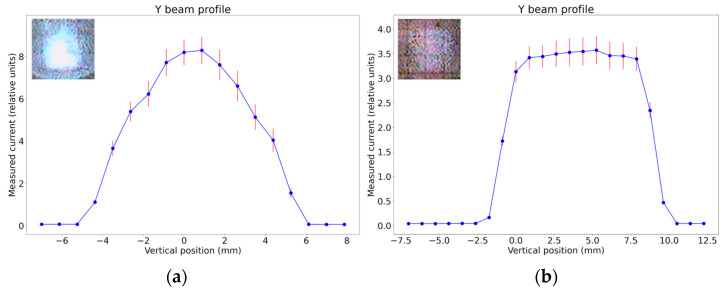
Results of BPM measurements for 10 MeV Fe^+4^ beam. Ion flux vertical profile: (**a**) for stationary ion beam, with the maximal opening of slits; (**b**) for scanning ion beam, with irradiation area width set by slits on 10 mm width. Insets are the luminescence image of each beam as seen on the quartz.

The homogeneity of ion flux is checked by a Beam Profile Monitor (BPM), placed at the sample manipulator. Examples of the beam profile measurements in the vertical direction for a stationary 10 MeV Fe beam and scanned 10 MeV Fe beam are shown in Figure 11. The beam profile in Figure 11a is shown for stationary beam and vertical slits open to the maximal extent, while in Figure 11b vertical slits were set to 10 mm width.

### 3.3. Stability of Sample Temperature

The stability of sample temperature during prolonged irradiations at DiFU has been tested by 24 h irradiation of Al_2_O_3_-coated steel sample by 12 MeV Au^+5^ ions of constant flux equal to 10^11^ ions/cm^2^s. Alumina-coated steel samples 10 mm long and 5 mm wide were 2 mm thick. They were coated by Al_2_O_3_ by Pulsed Laser Deposition [42] and Detonation Gun [43] methods. The thickness of the Al_2_O_3_ film was 1 µm.

**Figure 12:**
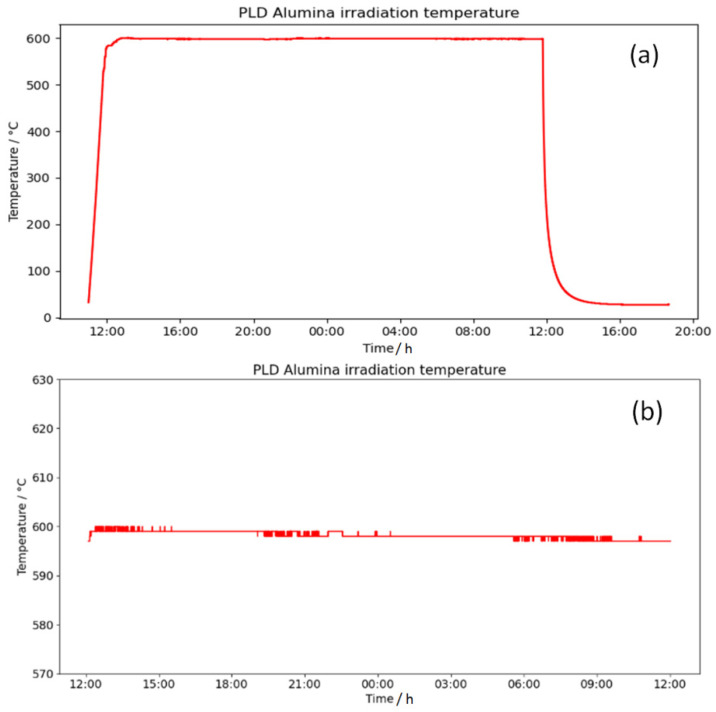
(**a**) Sample temperature is kept constant even in prolonged irradiation lasting over 24 h, shown here for irradiation of 24 h irradiation of Al_2_O_3_-coated steel sample by 12 MeV Au^+5^ ions of constant flux equal to 10^11^ ions/cm^2^. (**b**) Zoom-in of temperatures in the range of 550–650 °C, showing temperature variation of ± 1 °C.

Sample temperature over the entire irradiation time, including heating-up and cooling-down periods, was measured by two K-type thermocouples placed at the corners of the sample. As shown in Figure 12, the PID-controlled Tectra HC3500-LV heater power supply maintained stable sample temperature during irradiation, regardless of ion beam-induced sample heating with a variation of ± 1 °C.

### 3.4. Residual Gases’ Composition

**Figure 13:**
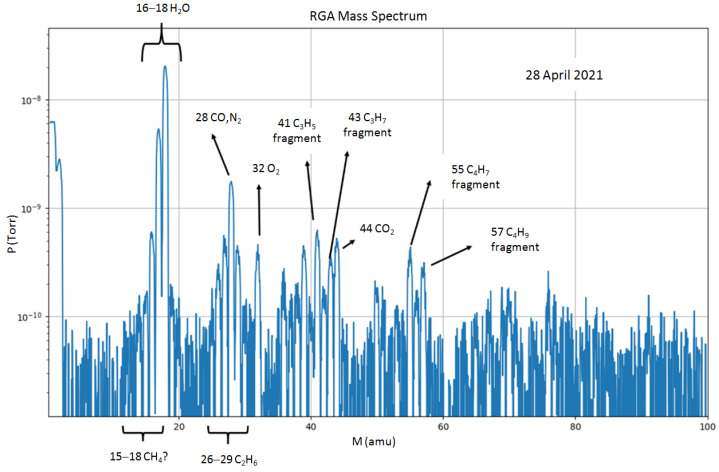
Identification of the peaks in the DiFU chamber residual gas spectrum.

RGA enables detailed insight into the composition of residual gasses’ within the DiFU chamber, as shown in Figure 13 for the DiFU chamber before plasma cleaning and without the application of LN2 cold traps.

**Figure 14:**
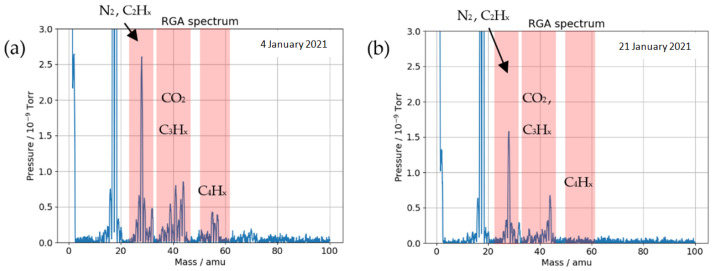
Residual gasses’ composition in DiFU chamber in: (**a**) intact chamber and (**b**) with all carbon mitigation procedures applied.

The effects of plasma cleaning of the chamber using oxygen plasma and utilization of LN2 cold traps during ion irradiation are shown in Figure 14, resulting in the reduction of key residual gas groups corresponding to C_3_H_X_ and C_4_H_x_ fragments. Small peaks of C_2_H_x_ fragments remain alongside reduced CO_2_ and N_2_.

## 4. Summary

The Dual-beam ion irradiation facility for FUsion materials (DiFU) enables:

- Irradiation of samples by single-and dual-ion beams on areas from 5 × 5 to 30 × 30 mm^2^;
- Electrostatic ion beam scanning;
- Adjustment of ion energy by foil degraders;
- Indirect ion beam profile/ion beam flux measurement and constant ion flux monitoring from slits;
- Three-dimensional sample positioning using a sample manipulator;
- Heating of sample and control of sample temperature by IR camera and thermocouples;
- Mitigated deposition of hydrocarbons on the irradiated area of the sample; and
- Installation of additional equipment brought or asked by users.

Aside from dual-beam irradiations by simultaneous heavy ion irradiation and proton or He ion implantation, both accelerators enable single beam irradiation sequentially i.e., heavy ion irradiation first, and proton or He ion irradiation later (or vice versa).

The DiFU facility was commissioned in 2019, with improvements introduced every year since. Particularly important are those following the ASTM E521—16: “Standard Practice for Investigating the Effects of Neutron Radiation Damage Using Charged-Particle Irradiation” [8].

## Data Availability

The data presented in this study are available on request from the corresponding authors.
